# Editorial: Novel approaches in cardiac imaging

**DOI:** 10.3389/fcvm.2023.1221927

**Published:** 2023-06-07

**Authors:** Francesco Pelliccia, Artur Dziewierz, Giuseppe Pannarale, Carlo Gaudio

**Affiliations:** ^1^ Department of Cardiovascular Sciences, University Sapienza, Rome, Italy; ^2^ 2nd Department of Cardiology, Institute of Cardiology, Jagiellonian University Medical College, Krakow, Poland

**Keywords:** cardiac imaging, echocardiography, computed tomography, magnetic resonance, artificial inteligence-AI

**Editorial on the Research Topic** Novel approaches in cardiac imaging

Over the past decade, advancements in cardiovascular imaging (1–3) have coincided with significant progress in diagnosing and treating various cardiac conditions (4–8). The implementation of speckle-tracking echocardiography now enables early detection of cardiac pathology while simultaneously reducing assessment variability between operators. Cardiac magnetic resonance has emerged as the gold-standard technique for non-invasively assessing biventricular dimensions, function, and myocardial tissue characteristics. Moreover, coronary computed tomography provides crucial information on plaque characteristics through non-invasive assessment of coronary arteries. Hybrid cardiac imaging combines various techniques and offers supplementary diagnostic and prognostic information. Last but not least, integrating artificial intelligence into modern cardiac imaging analysis promises a new frontier in diagnostic potential. Future technological advancements are expected to further emphasize the role of cardiovascular imaging in evaluating underlying pathophysiologic processes, thereby integrating pathophysiology, molecular medicine, and cardiovascular imaging.

This Special Issue delves into these significant aspects through 16 original research articles, an updated literature review, a systematic review and meta-analysis, and a case report. Here is a brief overview of these articles.

Chen et al. suggested a novel pipeline for labeling and imaging myocardial and vascular structures, which could be implemented in cardiac studies for examining heart structures at the single-cell level.

Guo et al. introduced a pioneering technology that combines diagnostic ultrasound with cyclic Arg-Gly-Asp-modified microbubbles targeting GP IIb/IIIa receptors. This approach can rapidly identify advanced atherosclerotic plaques and concurrently provide targeted therapy by dissolving activated and aggregated platelets.

El-Saadi et al. conducted a head-to-head comparison of myocardial strain using fast-strain encoding and feature tracking imaging in acute myocardial infarction. Both techniques exhibit acceptable agreement with two-dimensional echocardiography for longitudinal strain, with fast-strain encoding demonstrating higher sensitivity and specificity for detecting infarcted segments.

Zhang et al. evaluated left ventricular function in hypertrophic cardiomyopathy patients with preserved ejection fraction using left ventricular strain patterns derived by cardiac magnetic resonance feature tracking. The results suggest that left ventricular strain patterns are more sensitive than conventional ejection fraction for detecting subclinical cardiac dysfunction in hypertrophic cardiomyopathy. Also, strain patterns correlate with cardiac biomarkers, such as cardiac troponin and N-terminal prohormone of the brain natriuretic peptide.

Vattay et al. investigated the relationship between quantitative plaque metrics from coronary computed tomography angiography and segmental myocardial ischemia assessed using dynamic perfusion. Their findings suggest that total plaque volume is independently associated with myocardial blood flow but not with the severity of coronary stenosis.

Tang et al. explored the additional effects of mitral regurgitation on left ventricular strain impairment in patients with essential hypertension. Results showed that global peak strain was significantly more impaired in hypertensives with mitral regurgitation than in those without valvular insufficiency. Interestingly, the degree of mitral regurgitation was independently correlated with left ventricular global radial peak strain, circumferential peak strain, and global longitudinal peak strain.

Yu et al. assessed the diagnostic capability of real-time four-dimensional transesophageal echocardiography in detecting implant-related thrombus in patients who have undergone transcatheter left atrial appendage closure or have a cardiac implantable electronic device. The novel technique demonstrated a superior thrombus detection rate compared to conventional two-dimensional echocardiography. Furthermore, this real-time four-dimensional transesophageal echocardiography approach revealed distinct imaging features between a thrombus on an occluder and an electronic device.

Wang et al. investigated the utility of myocardial work parameters derived from pressure-strain loops in assessing cardiac function and predicting clinical prognosis in patients with pulmonary hypertension. The technique yielded two novel indices: right ventricular global constructive work and pulmonary arterial systolic pressure estimate. Both were predictive of clinical outcomes in patients with pulmonary hypertension.

Dejea et al. described the customization of an isolated, perfused heart system compatible with synchrotron-based x-ray phase contrast imaging. This innovative setup enables high-spatial resolution studies of heart architecture throughout the cardiac cycle, offering insights into the structural dynamics of the heart, including the effects of pharmaceuticals and other agents on the cardiac cycle.

Wang et al. compared longitudinal changes in color Tissue Doppler Imaging curves between normal controls and individuals at risk of future cardiac events. Noteworthy, they demonstrated that this technique could reveal an accelerated aging process in the hearts of individuals with cardiac risk factors. Consequently, they proposed Tissue Doppler Imaging as a valuable tool for identifying high-risk patients in a clinical setting.

Zhu et al. assessed fetal cardiovascular parameters using a two-dimensional speckle tracking technique to distinguish the size and systolic function differences in the left and right ventricles during pregnancy. They discovered that fetal cardiovascular physiology is characterized by a larger right ventricular volume (especially after 32 weeks) and greater left ventricular outputs, as indicated by higher ejection fraction, cardiac output, and stroke volume values.

Muraru et al. evaluated whether left ventricular volumes and ejection fraction measurements obtained by three-dimensional echocardiography offer any additional prognostic value over those acquired by two-dimensional echocardiography. Notably, in a total of 725 consecutive patients, left ventricular ejection fraction and end-systolic volume derived from three-dimensional echocardiography showed a stronger association with outcomes than the corresponding two-dimensional parameters.

Chen et al. proposed a novel method for labeling and imaging myocardial and vascular structures by using fluorescent dyes and transgenic markers. This innovative technique elucidates the three-dimensional morphology and spatial arrangement of cardiomyocytes and highlights structural differences in endothelial cells and capillaries. Overall, this new pipeline can define the structures of the entire heart at the single-cell level.

Beyls et al. investigated whether right ventricular longitudinal shortening fraction, a two-dimensional speckle tracking parameter used to assess right ventricular systolic function, yields consistent results regardless of whether it is assessed with conventional transthoracic echocardiography or transesophageal echocardiography. Crucially, the authors demonstrated that the values of the right ventricular longitudinal shortening fraction obtained with the two techniques are interchangeable.

Kusunose et al. applied a previously developed artificial intelligence model to predict heart failure and calculate the probability of elevated pulmonary artery wedge pressure. The findings showed the potential for using an artificial intelligence model on chest x-ray to predict pulmonary artery wedge pressure and its capacity to supplement prognostic information from other conventional clinical prognostic factors in heart failure. According to the authors of the study, these findings could enhance the accuracy of prediction models in heart failure, potentially leading to more informed clinical decision-making.

Erevik et al. aimed to determine the left ventricular response to increased exercise duration and intensity using novel echocardiographic tools to evaluate myocardial work and fatigue. They found that increased exercise intensity and duration during a cardiopulmonary exercise test and a 91-km mountain bike leisure race were associated with increased myocardial wasted work post-exercise, without changes in left ventricular ejection fraction and global longitudinal strain compared to baseline. These findings suggest that markers of myocardial inefficiency may precede a reduction in global left ventricular function, thus serving as early indicators of myocardial fatigue.

Yao et al. critically reviewed the benefits and limitations of four current ultrasound imaging modalities for assessing plaque vulnerability, considering the biological characteristics of the vulnerable plaque, and their value in clinical diagnosis, prognosis, and treatment efficacy assessment.

Sharma et al. conducted a systematic review and meta-analysis of the clinical application of longitudinal layer-specific strain as a diagnostic and prognostic instrument in ischemic heart disease. Their findings demonstrated that epicardial longitudinal layer-specific strain was the most significant diagnostic marker in patients with stable angina pectoris, whereas endocardial longitudinal layer-specific strain was the strongest diagnostic marker in patients with acute coronary syndrome. Furthermore, endocardial circumferential strain was a significant predictor of adverse outcomes in stable patients, while epicardial longitudinal layer-specific strain was the best predictor of outcomes in unstable patients.

Lin et al. presented a case of multiple biventricular aneurysms in arrhythmogenic cardiomyopathy, emphasizing that the pathological characteristics of arrhythmogenic cardiomyopathy can be better visualized using high-frequency linear ultrasound, with provides superior resolution.

In conclusion, future technological advancements, as discussed in this Special Issue, are expected to underscore the role of cardiovascular imaging in evaluating underlying pathophysiologic processes, thereby bridging the gap between pathophysiology, molecular medicine, and cardiovascular imaging (9, 10). Accordingly, multimodality imaging holds the promise of providing an unprecedented volume of diagnostic information, including an accurate depiction of coronary anatomy, atherosclerosis, myocardial ischemia, and tissue characterization. Such comprehensive data could enhance the diagnostic work-up and therapeutic options in real-world clinical practice.
